# Video game disorder and mental wellbeing among university students: a cross-sectional study

**DOI:** 10.11604/pamj.2022.41.89.31322

**Published:** 2022-02-01

**Authors:** Shaimaa Yaihya Abdel Raouf, Hala Marawan Gabr, Osama Al-Wutayd, Manal Ahmad Al-Batanony

**Affiliations:** 1 Public Health and Community Medicine Department, Faculty of Medicine, Menoufia University, Menoufia, Egypt,; 2 Department of Family and Community Medicine, Unaizah College of Medicine and Medical Sciences, Qassim University, Buraydah, Kingdom of Saudi Arabia

**Keywords:** Video game disorder, mental health, university students

## Abstract

**Introduction:**

video games are a popular adult pastime but have a potentially pervasive negative influence on gamers. The aim: was to determine the prevalence of video game disorder (VGD), its associated predictors, and its impact on the mental health of university students.

**Methods:**

a cross-sectional study was performed with a convenience sample of 2,364 undergraduate students. Sociodemographic criteria, the Internet Gaming Disorder-20 (IGD-20) questionnaire, and the Mental Health-5 (MH-5) questionnaire were used to collect data. The weights and heights of the students were self-reported. The average number of hours spent playing video games per week, the average number of hours of sleep per day, the favorite type of game played, and the main causes for playing were also included.

**Results:**

the prevalence of VGD among participants was 18.9%. The main predictors of VGD were being male, residing in an urban area, playing more hours per week, sleeping fewer hours per day, and having a higher body mass index, while having a low socioeconomic status was a protective factor. Mental health had a strong negative correlation with VGD. The types of games most frequently played by video game addicts were violent and action games. However, the most frequent reasons cited for playing were to improve one´s avatar, relaxation, and amusement.

**Conclusion:**

playing video games in moderation, adequate sleeping, and engaging in outdoor physical activities enhances mental well-being and physical functioning. Thus, it is critical to promote and encourage balanced, effective, stable approaches to video gaming among university students to maintain their mental well-being.

## Introduction

Video games are a complex type of interactive media that include gaming properties and necessitate active human-computer interaction [1]. Video gaming is a very popular pastime among adults. More than two billion users have been reported worldwide [2]. Most everyone now has easy access to technological devices from an early age, and those who use them find them fun and exciting [3]. Many factors, including photo-realistic graphics, increasingly sophisticated systems, and communicational characteristics, explain the popularity of video games [4]. The amount of time spent playing the latest generation of video games has increased as a result of such features. However, according to new World Health Organization mental health recommendations, screen time, including gaming, should be moderated [5]. In recent years, video gaming has quickly become the primary mode of entertainment for many people, and the global gaming industry has expanded at a rapid rate [6]. Every video game has unique characteristics that distinguish it from other video games, making some games more famous than others. Action, adventure, war, platform, role-playing, racing, shooter, simulation, sports, and strategy games are a few examples of types of video games [7]. The Diagnostic and Statistical Manual of Mental Disorders (DSM-5) and the International Classification of Diseases (ICD-11) both include videogame play as an addiction disorder [8, 9]. In the beta draft of the 11th Revision of the ICD, gaming disorder (GD) is characterized as a pattern of chronic or recurring offline and/or online gaming activity meeting three main clinical guidelines: (1) lack of control over gaming (e.g., timing, severity, rate, period, context, ending); (2) giving gaming a higher priority than other life preferences and everyday activities; and (3) continuing or worsening gaming despite adverse outcomes. GD can only be identified when the pattern of conduct is severe enough to cause a serious deterioration in intimate, social, family, academic, professional, or other essential areas of development [10].

From this perspective, although video gaming has been shown to improve concentration, multitasking, and executive function, it is also known to have costs when used excessively. Video game addicts are at risk of lower academic and vocational achievement, lower communication skills, and peer issues [11] in addition to worse mental well-being, sleeping habits, and overall health [12]. Surprisingly, according to the findings of a sociological survey conducted by the All-Russian Centre for the Study of Public Opinion, 77% of elderly respondents and 72% of youths consider video gaming to be a greater threat than the use of alcohol or antipsychotics [13]. As a result, an evaluation of the advantages and disadvantages of young people´s obsession with video games is needed. Much recent research has focused on the mental and psychological impacts of video games. For example, researchers have found that spending more time playing video games can lead to a lack of control and a strong impulse to play [14-17]. VGD has also been found to be related to aggression, anxiety, and depression as well as to personality traits such as a poorer social network, worse well-being, and low self-esteem [18, 19]. Moreover, gaming disorder has been shown to be associated with stress and maladaptive coping, having fewer real friends, worse mental well-being, and loneliness [20-22]. To the best of our knowledge, there has been a paucity of studies regarding VGD and mental health among young Egyptian adults. Thus, this study was conducted to determine the prevalence of VGD, its associated predictors, and its impact on mental health among university students in Egypt.

## Methods

**Study design and sampling:** a cross-sectional study was conducted between January 13 and February 2, 2021, using a convenience sample of Egyptian undergraduate students from both practical and theoretical colleges at Menoufia University. Inclusion criteria were any Egyptian student attending Menoufia University who agreed to participate in the study and had a social media account. All respondents participated voluntarily after being guaranteed anonymity. The study was exempted from written informed consent.

**Survey tool and data collection:** a Google form was used to create an online, self-administered, closed-ended questionnaire in Arabic. A link to the questionnaire was shared at random with participants on social media platforms (e.g., Facebook, WhatsApp) as well as personally with appropriate contacts of the investigators. The questionnaire consisted of an interface and three main parts containing a total of 41 questions. The questionnaire interface clearly explained the aim of the study.

The questionnaire´s three sections were as follows: 1) sociodemographic data, such as age in years, gender, residence, college type (practical or theoretical), student´s academic grade in the last year, stability of parents´ marriage, socioeconomic status, and number of family members. Other questions asked about the weight and height of the student, average number of hours of video games played per week, average number of hours of sleep per day, favorite type of game, and main reasons for playing; 2) the Arabic version of the Internet Gaming Disorder-20 (IGD-20) questionnaire was used to assess gaming activity during the past 12 months (whether played on a laptop or desktop computer or on any other device, either online or offline) [23]. The questionnaire is a self-assessment tool containing 20 statements measured on a five-point Likert scale ranging from 1 to 5 (strongly disagree to strongly agree). A respondent´s score was obtained by aggregating all 20 items. The higher the score, the worse the gaming disorder. The cut-off point for the IGD-20 test is 71 [24]. The tool has been validated by a Cronbach´s alpha coefficient of 0.92 [23]; 3) the mental health-5 (MH-5) questionnaire was used to evaluate the burden of video gaming on mental health in the last month. The tool included five questions on a six-point Likert scale ranging from 0 to 5 (always to never). The total score was calculated by adding and converting the five item scores into a number between 0 (the worst mental health) and 100 (the best mental health) [25]. The Cronbach´s alpha coefficient was 0.94, indicating good internal consistency [26]. All sections of the online survey had to be completed to be submitted; this prevented incomplete submissions. The sample size was calculated using Epi Info software with a 95% confidence interval, a 5% margin of error, and a 3.3% prevalence of problematic video gamers among adolescents and young adults [27]. The minimum sample size was estimated to be 2,299. At the time the survey closed, a total of 2,364 students had been recruited.

**Data management and analysis plan:** the Statistical Package for the Social Sciences (Version 25) was used to code and tabulate the data. The mean and standard deviation were summarized for quantitative data, or as a number and percentage for qualitative data. Comparison between quantitative variables were using the student t-test for measuring differences between means, and the chi-squared test was used to compare the qualitative variables for measuring association. A correlation test (r-test) was used to test associations between continuous data. Variables that were statistically significant (p < 0.05) in bivariate analysis were included in a multivariable regression analysis to assess the association of independent factors with the dependent factor (VGD). A p-value less than or equal to 0.05 was considered strong evidence against a null hypothesis.

## Results

**Prevalence of VGD:** among the university students studied, the prevalence of VGD was found to be 18.91% (Table 1).

**Table 1:** relation between video gaming disorder and different sociodemographic parameters among the university students studied

Participant characteristics	Total n = 2,364	Video Gaming Disorder		p-value
		Absent n (%)	Present n (%)	
		1,917 (81.1)	447(18.9)	
**Age in years**	20.20 ± 1.38	20.17 ± 1.38	20.29 ± 1.37	.1*
**Gender**				
Male	1,233	958 (77.7)	275(22.3)	< .001$
Female	1,131	959 (84.8)	172 (15.2)	
**SES**				
Low	44	44(100.0)	0(0.0)	.002€
Medium	1,936	1,640 (84.7)	296(15.3)	< .001€
High	384	234(60.9)	151(39.1)	< .001€
**College type**				
Theoretical	1,470	1,172 (79.7)	298(20.3)	.03$
Practical	894	745(83.3)	149(16.7)	
**Residence**				
Urban	1,326	1,024 (77.2)	302(22.7)	< .001$
Rural	1,038	893(86)	145(14.0)	
**Live with**				
Both parents	2,038	1,669 (81.9)	369 (18.1)	.01$
Single parent	326	248 (76.1)	78(23.9)	
BMI	25.72 ± 5.23	25.49 ± 4.82	26.24 ± 6.75	.006*
Hours played/week	18.99 ±7.95	17.71 ± 6.63	20.69 ± 8.35	< .001*
Hours slept/day	7.88 ± 0.87	7.83 ± 0.93	7.69 ± 0.75	.003*

* Student´s t-test, $Chi-squared test, ^€^Z-test

**Demographics of the subjects:** the ages of the subjects ranged from 18 to 22 years (20.20 ± 1.38). The distribution of females and males swas nearly equal (47.84% and 52.16%, respectively). In terms of place of residence, 1,326 (56.09%) of the respondents lived in an urban area, while 1,038 (43.91%) lived in a rural area. The vast majority of the group (81.90%) were of medium socioeconomic status, 16.24% (384 participants) were of high socioeconomic status, and only 1.86% (44 participants) were of low socioeconomic status. The percentage of students from theoretical colleges was 62.2% and from practical ones was 37.8% (Table 2).

**Table 2:** relation between video gaming disorder and parameters

Parameter	Video Gaming Disorder		p-value
	Absent n (%)	Present n (%)	
	1,917 (81.1)	447 (18.9)	
MH-5	85.11 8.73	83.92 9.76	.01*
**Academic achievement**			
Failed (n = 101)	82 (81.2)	19 (18.8)	.92
Passed (n = 579)	486 (83.9)	93 (16.1)	.05
Good (n = 717)	557 (77.7)	160 (22.3)	.006
Very good (n = 703)	490 (69.7)	113 (30.3)	.95
Excellent (n = 364)	302 (83.0)	62 (17.0)	.36
**Games frequently played**			
Massive multiplayer (n = 608) Simulation (n = 500)	466 (76.6) 396 (79.2)	142 (23.4) 104 (20.8)	.001 .25€
Adventure (n = 388)	323 (83.2)	65 (16.8)	.26
Strategy (n = 386)	317 (82.1)	69 (17.9)	.62
Action, shooter, or fighting (n = 345)	297 (86.1)	48 (13.9)	.01
Puzzles (n = 137)	118 (86.1)	19 (13.9)	.15
Reasons for playing: Relaxation (n = 1,274)	1,013 (79.5)	261 (20.5)	.03
Amusement (n = 752)	640 (85.1)	112 (14.9)	.001
Improving avatar (n = 187)	138 (73.8)	49 (26.2)	.01
Social relations (n = 43)	37 (86.0)	6 (14.0)	.52
Improving personal abilities (n = 108)	89 (82.4)	19 (17.6)	.82

* Student’s t-test, €Z-test

**VGD and sociodemographic parameters among the subjects:** VGD was significantly higher among males (p < .001), students from urban areas (p < .001), subjects attending theoretical colleges (p = .03), subjects living with a single parent (p = .01), and subjects with high and medium socioeconomic statuses (p < .001). VGD was more prevalent among respondents with a higher body mass index (BMI) (p = .006), those who played more hours per week (p < .001), and those who slept fewer hours per day (p = .003) (Table 1).

**VGD, mental health, and academic achievement:** the mean value of the MH-5 questionnaire measuring mental health was significantly lower among students with VGD (83.92 ± 9.76) than among their counterparts (85.11 ± 8.73, p = .01). VGD had a negative effect on academic achievement, with video game addicts being lower achievers than other respondents (p = .006). A significant relationship was observed between VGD and the types of games played frequently. Violent, aggressive, and action games, such as massive multiplayer, adventure, shooter, and fighting games, were the types most frequently reported by video game addicts (p < .001 and 0.01, respectively). Regarding the reasons behind playing, improving one´s avatar, relaxation, and amusement were the most frequently cited reasons by disordered gamers (p = .01, .03, and <.001, respectively) (Table 2).

**Correlation between VGD and various parameters:** a significant positive correlation was observed between VGD and the number of hours played per week (r = .05, p = .008), socioeconomic status (r = .28, p < .001), and BMI (r = .23, p < .001). Meanwhile, there was a significant negative correlation between VGD and mental health status (r = -.06, p = .003) and number of hours of sleep per day (r = -.55, p < .001) (Table 3).

**Table 3:** correlation between video gaming disorder score and different parameters

Parameter	VGD score	
	r-test	p-value
BMI	.23	< .001
SES	.28	< .001
Hours slept/day	-.55	< .001
Hours played/week	.05	.008
Mental health status (MH-5)	-.06	.003

**Main predictors of VGD:** a multivariable regression analysis revealed that the main predictors of VGD were being male (OR = 1.79, 95% CI [1.09, 1.72]), living in an urban area (OR = 8.73, 95% CI [3.56, 21.41]), playing more hours per week (OR = 1.21, 95% CI [1.17, 1.24]), and having a higher BMI (OR = 1.14, 95% CI [1.096, 1.185]). Moreover, it was observed that sleeping more hours per day (OR = 0.32, 95% CI [0.26, 0.39]) and having a low socioeconomic status (OR = 0.38, 95% CI [0.21, 0.66]) were the main protective factors against VGD (Table 4).

**Table 4:** multivariable analysis for independent predictors of video gaming disorder

Participant characteristics	OR	95% CI	p-value
Sex			
Female (reference)			
Male	1.79	1.09-1.72	< .001
College type			
Practical (reference)			
Theoretical	0.70	0.27-1.81	.46
SES			
Low SES	0.38	0.21-0.66	.04
Medium SES (reference)	1.09	0.45-2.61	.86
High			
Residence			
Rural (reference)			
Urban	8.73	3.56-21.41	< .001
Parent status			
Both parents (reference)	0.62	0.34-1.12	.11
Single parent			
Hours played/week	1.21	1.17-1.24	< .001
Hours slept/day	0.32	0.26-0.39	< .001
BMI:	1.14	1.1-1.19	< .001

**Ethical considerations:** the Institutional Review Board of the Faculty of Medicine at Menoufia University in Egypt approved the ethics of this study before data were collected.

## Discussion

The current study revealed that the prevalence of VGD among the university students studied was 18.91%. This high percentage reflects the popularity of gaming among youths as well as its accessibility. This finding is consistent with the reported prevalence among university students in Ghana (12.2%), adolescents in Hong Kong (15.6%), and expatriate adolescents in Saudi Arabia (15.8%) [19, 28, 29]. In this study, male students had a significantly higher prevalence of video gaming addiction than their female counterparts. This result is congruent with a recent study in Ghana, in which most video gamers were male [28]. Other studies have also reported that males were more likely to be video game addicts than females [19,30]. This study observed a higher prevalence of VGD in students living in urban areas and with medium and high socioeconomic statuses. A correlation test revealed that VGD was positively correlated with medium and high socioeconomic statuses. Similarly, Shi *et al*. [31] found that students living in urban regions experienced significantly more problematic video gaming than those from non-urban regions. VGD and its relationship with socioeconomic status remains somewhat controversial. In contrast to our results, Choo *et al*. [32] and El-Nahas *et al*. [33] reported that problematic gaming disorder was negatively associated with high socioeconomic status. Wang *et al*. [29] pointed to an absence of any association between gaming addiction and socioeconomic status. Our finding could be explained by the fact that one needs a reasonably high income level to purchase the modern mobile devices and consoles required for gaming. Purchasing fast internet and gaming software also requires a medium-to-high economic level. Worldwide game software sales were expected to grow at a compound annual growth rate (CAGR) of 11.0% between 2021 and 2023 to reach $223 billion [34]. Our study revealed that VGD was significantly prevalent among students attending theoretical colleges and those living with a single parent. The first finding is supported by a previous Egyptian study of university students [33]. Consistent with our result, Rehbein and Baier [35] found that a student living in a single-parent family was at risk of becoming a disordered gamer. Our study revealed that a high BMI was also significantly associated and positively correlated with VGD. This finding is congruent with that of Saquib *et al*. [19] who reported that being overweight was significantly associated with gaming addiction. Similarly, the previous literature has classified video gaming as a sedentary action and argued that, in recent decades, combined with poor dietary habits, it can lead to a troubling increase in obesity in children and youths [36-38]. In this study, the more time spent gaming per week and the fewer hours slept per day, the higher the frequency of VGD. The first finding is in line with Miezah *et al*. [28] and Wang *et al*. [29] while the second result agrees with the results of Saquib *et al*. [19] and Rehbein *et al*. [39]. In evaluating the mental health of subjects, it was obvious that students with VGD had a significantly lower mean value than their counterparts. Many studies have addressed the burden of VGD on mental and psychological health. In a nationwide German survey, Rehbein et al. [39] found that diminished leisure activities, high truancy rates, and extreme suicidal ideation were related to VGD. In the U.S., Tortolero *et al*. [40] reported an increased frequency among preadolescents of depression manifestations and more hours spent playing violent video games. Furthermore, Mérelle *et al*. [41] linked suicidal ideation, as a mental health disorder, to VGD. Broadly speaking, Andreassen *et al*. [42] revealed a strong association between the addictive behavior of video gaming and many mental health problems, including anxiety, depression, obsessive compulsive disorder, and attention-deficit/hyperactivity disorder. Long periods of social isolation and technology use due to video gaming have the potential to cement an unhealthy lifestyle [43]. This study revealed a negative relationship between VGD and academic achievement, where low achievers had a higher risk of becoming gaming addicts. This is supported by evidence from epidemiological studies [39, 44, 45]. In our study, disordered gamers frequently played violent, aggressive, and action games, such as massive multiplayer, adventure, shooter, and fighting games. Similarly, it has been reported that highly violent video games and action games, like racing and adventure games, were the types played most often by problematic video gamers [40, 46]. Among problematic gamers in our study, the main reasons cited for playing video games were to improve one´s avatar, relaxation, and amusement. Associations between gaming disorder and escapism have been found by other authors [47, 48]. In addition, a multivariable regression analysis found an important independent relationship between VGD and being male, living in an urban area, the number of hours spent playing each week, sleeping fewer hours per day, high socioeconomic status, and increased BMI. Correspondingly, Wittek *et al*. [30] found that male respondents had a higher risk of addiction than female respondents, according to both crude and adjusted analyses. Moreover, Ferreira *et al*. [49] highlighted that playing video games non-stop for hours is a significant predictor of gaming disorder.

There were some notable limitations to our study. The use of a cross-sectional sample prevented a causality analysis, so an interpretation of the regression results may be of value. Using a convenience sample, as opposed to a random sample, can cause selection bias, lowering internal validity. Recall bias could also be a factor due to the use of online self-reported questionnaires, which were influenced to some degree by the students´ integrity and recall capacity. The findings of the study are not generalizable to the study population. Despite these limitations, we remain confident that the findings of this study will offer insights and data on VGD and its consequences for university students´ mental well-being.

## Conclusion

About one-fifth of the university students studied experienced VGD, which was strongly linked to being male, living in an urban area, having a high socioeconomic status and higher BMI, playing more hours per week, and sleeping fewer hours per day. VGD negatively impacted these subjects´ mental health. Accordingly, education and health authorities should view video game use and/or addiction as a health threat. Programs should be created to help people manage problematic video gaming. University faculty must continue to track and help students meet their psychosocial needs by detecting vulnerable students early. Future empirical longitudinal studies should address how to deal with VGD alone or in combination with other types of addiction, such as substance abuse.

### What is known about this topic

- Worldwide, adult video gaming is an extremely popular pastime;
- In today´s culture and from a young age, almost everyone has easy access to technical devices, and those who utilize them find them enjoyable and engaging;
- Video gaming has some benefits, such as improving concentration, but it also has adverse effects.

### What this study adds

- VGDs appeared to become more prevalent among university students;
- Excessive video game use, insufficient sleep, and a lack of participation in outdoor physical activities are all detrimental to youth´s mental and physical health;
- To sustain university students' mental health, it is vital to promote and encourage balanced, effective, and steady approaches to video gaming.
